# Locomotor skill acquisition in virtual reality shows sustained transfer to the real world

**DOI:** 10.1186/s12984-019-0584-y

**Published:** 2019-09-14

**Authors:** Aram Kim, Nicolas Schweighofer, James M. Finley

**Affiliations:** 1 Division of Biokinesiology and Physical Therapy, University of Southern California, 1540 E. Alcazar St, CHP 155, Los Angeles, CA 90033 USA; 2 Neuroscience Graduate Program, University of Southern California, Los Angeles, CA 90089 USA; 3 Department of Biomedical Engineering, University of Southern California, Los Angeles, CA 90089 USA; 4 Department of Computer Science, University of Southern California, Los Angeles, CA 90089 USA

**Keywords:** Obstacle negotiation, Virtual reality, Motor learning, Transfer, Retention

## Abstract

**Background:**

Virtual reality (VR) is a potentially promising tool for enhancing real-world locomotion in individuals with mobility impairment through its ability to provide personalized performance feedback and simulate real-world challenges. However, it is unknown whether novel locomotor skills learned in VR show sustained transfer to the real world. Here, as an initial step towards developing a VR-based clinical intervention, we study how young adults learn and transfer a treadmill-based virtual obstacle negotiation skill to the real world.

**Methods:**

On Day 1, participants crossed virtual obstacles while walking on a treadmill, with the instruction to minimize foot clearance during obstacle crossing. Gradual changes in performance during training were fit via non-linear mixed effect models. Immediate transfer was measured by foot clearance during physical obstacle crossing while walking over-ground. Retention of the obstacle negotiation skill in VR and retention of over-ground transfer were assessed after 24 h.

**Results:**

On Day 1, participants systematically reduced foot clearance throughout practice by an average of 5 cm (SD 4 cm) and transferred 3 cm (SD 1 cm) of this reduction to over-ground walking. The acquired reduction in foot clearance was also retained after 24 h in VR and over-ground. There was only a small, but significant 0.8 cm increase in foot clearance in VR and no significant increase in clearance over-ground on Day 2. Moreover, individual differences in final performance at the end of practice on Day 1 predicted retention both in VR and in the real environment.

**Conclusions:**

Overall, our results support the use of VR for locomotor training as skills learned in a virtual environment readily transfer to real-world locomotion. Future work is needed to determine if VR-based locomotor training leads to sustained transfer in clinical populations with mobility impairments, such as individuals with Parkinson’s disease and stroke survivors.

## Background

In recent years, virtual reality (VR) has been increasingly used to provide engaging, interactive, and task-specific locomotor training [1–8]. These studies have simulated walking in different environments such as parks or streets [3, 4], walking on a slope [3], or walking while avoiding obstacles [3–5, 7]. VR-based locomotor training frequently includes obstacle negotiation because it is an essential locomotor skill in the community [4, 5, 7] and tripping over obstacles is a common cause of falls in many patient populations [9]. The clinical application of VR-based training interventions is predicated on the idea that practice in VR will lead to lasting changes in trained skills and that these changes will influence real-world behavior. Therefore, understanding how locomotor skills acquired in VR are retained and how these skills generalize to the real world is critical for determining the long-term utility of VR for locomotor rehabilitation.

The presence of lasting changes in a motor skill as a result of practice is a hallmark of motor learning and this retention process has been examined across a wide variety of real and virtual learning contexts. Retention of motor skills has been examined in response to VR training, particularly in fields such as flight and medical procedural training. For example, complex surgical and medical skills are performed faster and more accurately during a retention session following a single day of VR-based training [10–13]. Retention of locomotor skills is often explored in studies that analyze how people adapt to external perturbations such as a split-belt treadmill which has separate belts for the right and left legs [14–16], elastic force fields [17], robotic exoskeletons [18], or added loads [19]. For instance, studies of split-belt treadmill adaptation have revealed that the increases in step length asymmetry observed during initial exposure to the belts moving at different speeds significantly decreased with subsequent exposures to the device [14–16]. A recent study by Krishnan and colleagues also investigated locomotor skill learning during a tracking task in which participants were instructed to match a pre-defined target of hip and knee trajectories as accurately as possible during the swing phase of the gait [20]. They found that the reduction in tracking error achieved through practice is retained the following day. Although motor skill learning in VR and locomotor learning have been examined in isolation, it remains to be seen how locomotor skills are acquired and retained following training in a virtual environment.

Skill transfer, which is defined as “*the gain or loss in the capability for performance in one task as a result of practice or experience on some other task*” [21], is another key feature of motor learning. Skill transfer is particularly critical when skill acquisition occurs in a context that differs from the environment in which the skill is to be expressed. One way in which skill transfer has been evaluated during motor learning is by measuring how the adaptation of reaching in a robot-generated force field generalizes to unconstrained reaching. This work has shown that adaptation to reaching in a curl-field leads to increased curvature during reaching in free space [22, 23]. Moreover, studies of treadmill-based locomotor skill learning often evaluate transfer of learned skills from treadmill walking to over-ground. For example, during split-belt treadmill adaptation, the learned changes in interlimb symmetry partially transfer to over-ground walking [24]. Further, VR-based training of obstacle negotiation on a treadmill led to increased walking speeds in the lab [5, 7] and community [4]. However, the evaluation of transfer in these VR-based training studies was based on outcome measures such as walking speed that did not reflect the objective of the training task, which was the control of foot clearance obstacle negotiation. Therefore**,** it remains to be seen if the elements of skill from VR-training transfer to over-ground walking.

Underlying individual differences in learning can influence motor skill retention and transfer to new environments. For example, a recent study demonstrated that healthy older adults and people post-stroke who acquire a motor sequence skill at a faster rate also show greater retention of that skill [25]. Similarly, the rate of skill acquisition for a reaching task during early training predicts faster trial completion time at 1-month follow-up [26]. Lastly, the magnitude of improvements in reaching speed during skill acquisition predicts long-term changes in reaching speed in healthy individuals [27]. Studies of individual differences in transfer have most often sought to understand how the practice of a skill with one limb influences performance of the same skill with the untrained limb. For example, interlimb transfer of motor skills acquired through visuomotor adaptation varies with handedness [28] and individual differences in baseline movement variability [29]. However, far less work has sought to understand how individual differences in skill acquisition affect the transfer of learned skills to new environments. Overall, the influence of individual differences in skill acquisition on locomotor skill retention and sustained transfer has yet to be determined.

Here, we determined how individual differences in locomotor skill learning during virtual reality treadmill-based training influence retention and transfer of learned skills to over-ground walking in the real world. We used a VR-based version of a previously established precision obstacle negotiation task [30, 31] and asked 1) whether healthy young adults could learn to minimize clearance during virtual obstacle negotiation, 2) if the learned skill transferred to over-ground walking, 3) if the learned skill was retained in both VR and the real world after 24 h, and 4) if individual differences in the amount or rate of skill acquisition could predict retention and transfer. We hypothesized that 1) participants would reduce foot clearance in VR during practice on Day 1 and that 2) the reduced foot clearance in VR would transfer to over-ground obstacle negotiation. We also hypothesized that 3) the reduction in foot clearance in VR and over-ground would be retained in each environment after a 24-h retention period. Lastly, given that the rate and magnitude of the performance improvement during skill acquisition have been established as predictors of skill retention in previous studies, we also hypothesized that 4) these measures would predict retention of the learned skill in VR and over-ground. Given the growing use of VR for motor skill learning, our results may provide a unique opportunity to understand the factors that influence how training in VR might lead to long-term improvements in skilled locomotion.

## Methods

### Participants

Nineteen healthy young adults participated in the study (10 female, average age of 26 ± 4 years). All participants had normal vision or corrected-to-normal vision. Study procedures were approved by the Institutional Review Board at the University of Southern California and all participants provided written, informed consent before testing began. All aspects of the study conformed to the principles described in the Declaration of Helsinki.

### Experimental protocol

**Fig. 1:**
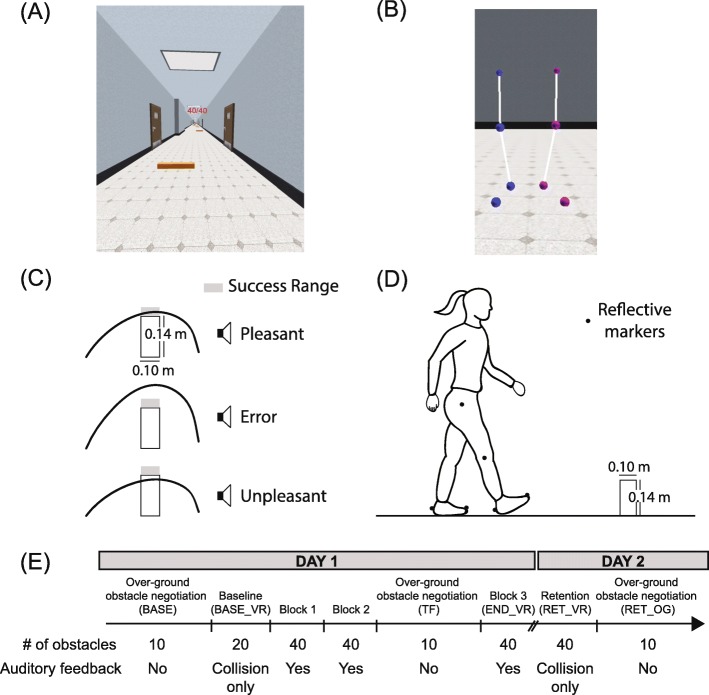
Experimental setup and protocol. **a** Virtual corridor with obstacles and an eye-level display of participants’ current score. **b** Visual feedback of the lower extremities viewed from a third-person perspective. Spheres represent the position of markers placed on the lower extremities. Line segments connecting the spheres were used to provide a visual representation of limb segment length. During the study, participants viewed the representation of the lower extremities from a first-person viewpoint. **c** Schematic diagram of the mapping between the participant’s performance and the auditory feedback they received. **d** Over-ground obstacle negotiation setup. **e** Experimental protocol illustrating the day of the study, the trial type, number of obstacles per trial, and whether auditory performance feedback was provided

Participants completed a VR-based version of a previously established obstacle negotiation task [30, 31] where they were instructed to minimize foot clearance when crossing the obstacle. This task was specifically chosen because it allowed us to examine a form of skill acquisition which requires participants to learn a precise mapping between the perception of the spatial location of virtual obstacles and the control of foot trajectory. Participants walked on a treadmill (Bertec Fully Instrumented Treadmill, USA) while wearing a head-mounted display (HMD) and interacting with the virtual environment. The velocity of the virtual environment was synchronized with the treadmill at 1.0 m/s, and an IMU within the HMD controlled the orientation of the viewpoint. The virtual simulation was run at 60 Hz and the motion capture system had a real-time delay of 3.5 ms. All participants were instructed to lightly hold on to a handrail while walking on the treadmill. Participants viewed the environment (Fig. 1a) and the virtual representation of their legs from a first-person perspective (Fig. 1b). Their body was represented by a set of spheres located at the hip, knee, heel, and toe, bilaterally and lines connecting the spheres to represent the limb segments [32]. Marker placement details are described below in the *Data Collection and Processing* section.

The virtual environment consisted of a corridor with obstacles (Fig. 1a) and was developed using Sketchup (Trimble Navigation Limited, USA). The interaction between participants and the virtual environment was controlled using Vizard (WorldViz, USA). A total of 40 virtual obstacles, 20 each on the right and left side, were randomly placed along the corridor at intervals of between 5 m and 10 m to provide sufficient space for participants to fully recover their typical gait pattern after crossing the previous obstacle. Previous work has also established that a distance of 2 m between obstacles is sufficient for people to cross each obstacle as if it was independent of the others [33]. The height of the obstacles was adapted from the previous study, which used the same objective of minimizing foot clearance, but with a physical obstacle [34]. The obstacles were 0.14 m in height and 0.10 m in depth. The placement of the obstacles was lateralized so that participants either crossed the obstacle with the right or left leg. In VR, the mediolateral distance between the feet was constrained such that participants only saw movement of their legs in the sagittal plane. We imposed this constraint to ensure bilateral obstacle negotiation. An Oculus Rift Development Kit 2 HMD was used to display the virtual environment. The HMD had a 100-degree horizontal and vertical field of view, a resolution of 960 X 1080 pixels for each eye, a mass of approximately 450 g and 100% binocular overlap.

The study consisted of two visits on consecutive days (Fig. 1e). On Day 1, participants first walked over-ground while stepping over a single physical obstacle ten times without the HMD (BASE, Fig. 1d). If there was a collision during physical obstacle negotiation, participants repeated the trial. Then, they moved to the treadmill and donned the HMD. For the first treadmill trial, they walked while stepping over 20 obstacles while receiving auditory feedback in the form of an unpleasant sound following collisions with the obstacles (BASE_VR). Following BASE_VR, participants had three practice blocks of obstacle negotiation in VR and each block consisted of 40 obstacles. Between blocks 2 and 3 we evaluated immediate transfer by having participants perform a physical obstacle negotiation task ten times over-ground without the HMD (TF). In all trials, participants were instructed to cross each obstacle using only the limb ipsilateral to the obstacle and to minimize the vertical distance between the foot and the obstacles during the crossing. During the over-ground obstacle negotiation task, we instructed participants to use either the right or left leg to step over the obstacle prior to walking. The right-left order of the ten trials was randomized for each participant. Moreover, for over-ground walking, participants were instructed to maintain their walking speed throughout obstacle negotiation and avoid slowing down during the approaching and crossing step. During the training blocks, participants received three types of auditory performance feedback: 1) a pleasant sound when foot clearance was within a target range of 0–2 cm, which was used to be consistent with previous studies [30, 31], 2) an error sound whose frequency scaled with foot clearance when clearance was greater than 2 cm, and 3) a failure sound following collisions with the obstacle (Fig. 1c). Participants began each trial with 40 points and lost one point for each collision with the obstacle. After 24 h, participants returned to the laboratory and completed one retention block of 40 obstacles in VR with no auditory feedback other than collision feedback (RET_VR). We then assessed over-ground retention (RET_OG) in the same manner as BASE.

### Data collection

Participant’s lower extremity kinematics were tracked using infrared-emitting LEDs (Qualisys, Sweden) placed on the following landmarks bilaterally: toe (approximately the second toe), heel, lateral femoral epicondyle, and greater trochanter. During virtual obstacle crossing, the vertical distance between the obstacle and both the toe and heel markers was calculated throughout the crossing step based on the raw marker position data, and the lowest value between the two markers was used as our measure of foot clearance. For over-ground obstacle trials, marker positions were recorded at 100 Hz in Qualisys Track Manager and were post-processed with a 4th order Butterworth low pass filter with a cutoff frequency of 6 Hz. Here, the measure of foot clearance was the same as trials during virtual obstacle crossing.

### Statistical analysis

The change in foot clearance during baseline and training trials was modeled using a nonlinear, exponential mixed-effects (NLME) model to capture the exponential time course of learning [35] and individual differences in the initial and final performance. The advantage of using NLME models over individual exponential fits is that NLME models can provide more precise parameter estimates and explain more variance than individual exponential fits [36]. Moreover, NLME models also capture fixed effects that are common across individuals. Data from the baseline trial were included because participants were instructed to minimize foot clearance during this session and exhibited improvements in performance over the trial. The NLME model consisted of an exponential decay term to capture the reduction in foot clearance during acquisition and constants that captured initial performance and the performance plateau (Eq. 1):

1 $$ {\hat{FC}}_{i,j}={A}_i\times {e}^{\frac{-j}{tau_i}}+{D}_i $$

Here, ${\hat{FC}}_{i,j}$ was the estimated foot clearance for each participant (*i* = 1:19) and each obstacle (*j* = 1:N) where N is the number of obstacles that were crossed successfully (maximum: 140). *A*_*i*_ represents the approximate reduction in foot clearance over the course of practice for our sample. *tau*_*i*_ represents the individual acquisition rate. Only obstacles crossed without collisions were included in the model because foot clearance during collisions would result in negative values. ${\hat{FC}}_{i,j}$, *A*_*i*_*,* and *D*_*i*_ were expressed in meters and *tau*_*i*_ was expressed in units of obstacles. Model parameters were estimated using NLME fit with stochastic Expectation-Maximization algorithm function, *nlmefitsa,* from MATLAB R2017a (Natick, MA). The sum of the fixed and random effects from these models represented participant-specific effects. R^2^ values were used to measure the goodness of fit of the final models. There was no significant difference in foot clearance during skill acquisition between the left and right legs (t(184) = − 0.17, *p* = 0.87). Therefore, we analyzed all obstacles together in a single ensemble.

The definitions of all dependent variables that were derived from the NLME model and measured foot clearance are found in Table 1. We estimated initial and final clearance, the relative and absolute amount of skill acquisition, and lastly, acquisition rate. Together, these variables were used to test how skill acquisition and performance during practice related to retention. Initial clearance represented the estimated foot clearance at the first obstacle. Final foot clearance was estimated by summing the amplitude of the exponential term at the end of practice and the asymptote. The absolute amount of skill acquisition was estimated from the model as the change in clearance from the first obstacle to the last obstacle. The relative amount of skill acquisition represented the change in performance from each individual’s initial foot clearance to final foot clearance normalized by the initial foot clearance and expressed as a percentage.

**Table 1:** Dependent variables derived from the NLME model

Variable	Definition
***A***_***i***_ ***+ D***_***i***_	Initial foot clearance
${\boldsymbol{A}}_{\boldsymbol{i}}\times {\boldsymbol{e}}^{\frac{-\boldsymbol{N}}{{\boldsymbol{tau}}_{\boldsymbol{i}}}}+{\boldsymbol{D}}_{\boldsymbol{i}}$	Final foot clearance
${\boldsymbol{A}}_{\boldsymbol{i}}-{\boldsymbol{A}}_{\boldsymbol{i}}\times {\boldsymbol{e}}^{\frac{-\boldsymbol{N}}{{\boldsymbol{tau}}_{\boldsymbol{i}}}}$	Absolute amount of skill acquisition
***tau***_***i***_	Rate of skill acquisition
$\frac{{\boldsymbol{A}}_{\boldsymbol{i}}-{\boldsymbol{A}}_{\boldsymbol{i}}\times {\boldsymbol{e}}^{\frac{-\boldsymbol{N}}{{\boldsymbol{tau}}_{\boldsymbol{i}}}}}{{\boldsymbol{A}}_{\boldsymbol{i}}+{\boldsymbol{D}}_{\boldsymbol{i}}}\times \mathbf{100}$	Relative amount of skill acquisition

i: Participant ID, N: The number of successful clearances on the treadmill

We quantified the absolute magnitude of transfer to over-ground walking on Day 1 (absolute transfer) as the difference in foot clearance between the TF and BASE blocks. Each of these metrics was computed as the average foot clearance over the ten obstacles during each trial. We conducted an additional analysis using a linear mixed-effects model with a fixed effect of trial and a random intercept for each participant to test whether there were any within-block changes in clearance over the ten obstacles. We also quantified the fraction of the improvement in skill in VR that was transferred to over-ground walking on Day 1 (relative transfer) as the ratio of absolute transfer to the amount of skill acquisition estimated from the NLME model expressed as a percentage. Foot clearance during RET_VR was computed as the average foot clearance over all obstacles after removing obstacle crossings where collisions occurred. Retention of the locomotor skill in VR on Day 2 was calculated as the difference in foot clearance between RET_VR and END_VR where each of these measures was the average of all successful obstacle crossings in each block. Similarly, over-ground retention was computed as the difference in clearance between RET_OG on Day 2 and TF on Day 1. Because the goal of the task was to reduce foot clearance, more negative values indicated a larger improvement. Foot clearance during over-ground trials was averaged across ten trials.

Dependent variables calculated for absolute transfer, relative transfer, and over-ground retention were tested for normality using the Lilliefors test in MATLAB. If the variables satisfied the normality test, single-sample t-tests were performed to test whether participants transferred the obstacle negotiation skill to over-ground walking, whether they showed relative transfer during over-ground walking, and whether they retained the reduction in foot clearance in VR and over-ground on Day 2. If the variables did not satisfy the normality test, one-sample Wilcoxon signed-rank tests were performed instead.

We also used multiple linear regression to determine whether the amount or rate of locomotor skill acquisition during practice predicted retention of the obstacle negotiation skill in VR and over-ground on Day 2, respectively. Specifically, we hypothesized that the relative amount and rate of skill acquisition would predict retention in VR, similar to what has been observed in previous studies [25–27]. The set of predictors for retention in VR included final foot clearance and the absolute and relative amount of skill acquisition from Eq. 1. The predictors for over-ground retention included each of these predictors, foot clearance during BASE and TF, and the change in over-ground foot clearance on Day 1. The predictors included in each regression model were tested for multicollinearity using the Variance Inflation Factor (VIF). If the predictor had VIF higher than 5, which indicates that the predictor was highly correlated with other predictors, that predictor was removed from the model. After removing collinear variables, we used the best subset selection method for variable selection and selected the model with the lowest Bayesian Information Criterion (BIC) [37]. The alpha level was set at *p* < 0.05. All statistical analyses were done in MATLAB.

## Results

### Acquisition and transfer of skilled obstacle negotiation

**Fig. 2:**
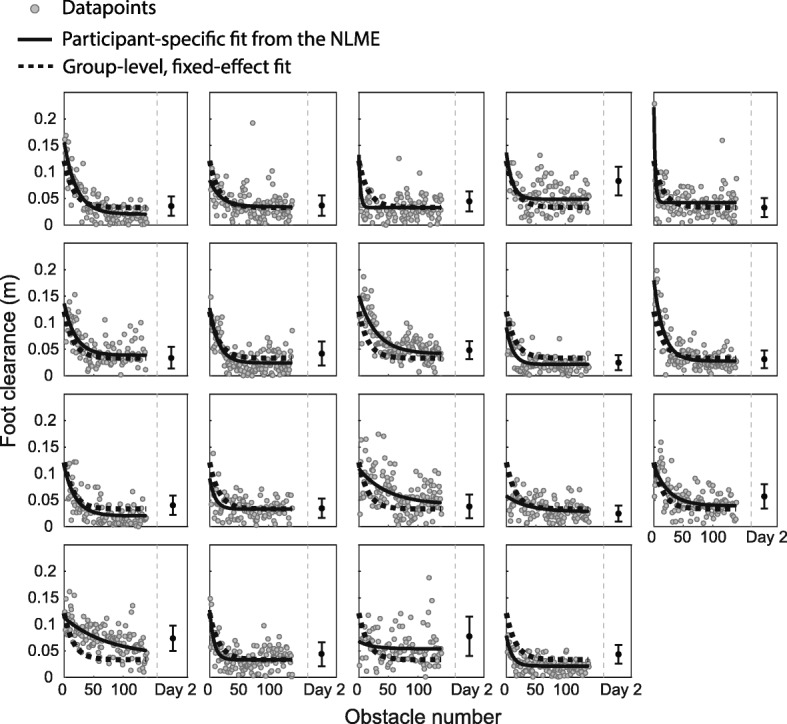
Individual foot clearance data and fit from the NLME model. Gray points represent foot clearance during each obstacle crossing in VR on Day 1, the black curve represents the participant-specific fit from the NLME, and the dashed curve represents the group level fit of the NLME. The black points and error bars after the gray dashed vertical line represent the average and standard deviation of foot clearance in VR on Day 2, respectively

**Fig. 3:**
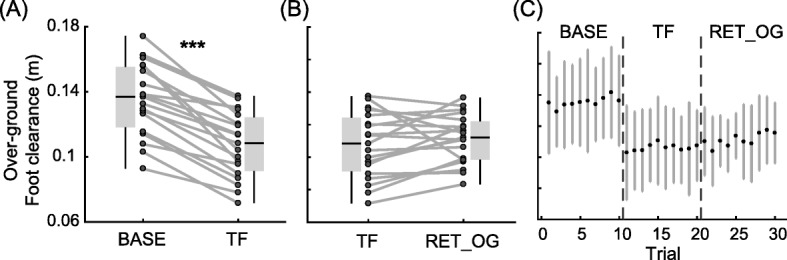
Over-ground transfer on Day 1 and Day 2. **a** Transfer to over-ground walking on Day 1. Here, reductions in foot clearance indicate improvements in skill. **b** Over-ground retention on Day 2. Horizontal lines within each box indicate median values and the bottom and top boundaries of each box indicate the 25th and 75th percentiles. Dark gray points represent individual data points and the gray lines connecting the points represent the change in foot clearance across trials. **c** Trials during over-ground obstacle negotiation. Black points represent average foot clearance across all participants and gray vertical lines represent standard deviation. BASE: baseline block for over-ground on Day 1, TF: transfer block for over-ground on Day 1, and RET_OG: retention block for over-ground on Day 2. The asterisks (***) indicate statistically significant differences from zero at *p* < 0.001

All participants successfully reduced their foot clearance throughout practice on Day 1 (Fig. 2). The average R^2^ for the NLME model was 0.33 ± 0.22 (SD). The average of the exponential amplitude parameter *A*_*i*_ was 0.09 ± 0.04 m. The average time constant *tau*_*i*_ was 21 ± 18 obstacles, which indicated that, on average, individuals needed to successfully negotiate 21 obstacles to achieve 66% of their total reduction in clearance. Lastly, the average of the constant *D*_*i*_ was 0.04 ± 0.01 m. Based on our parameter estimates, foot clearance during virtual obstacle negotiation in VR was reduced by 69 ± 15% during the acquisition period on Day 1. The average number of collisions was 3 ± 3 out of 20 obstacles and 20 ± 10 out of 120 obstacles in BASE_VR and practice blocks, respectively. Practice in VR led to a transfer of reductions in foot clearance to over-ground walking at the end of Day 1 (Fig. 3a). The average walking speed over-ground was 0.9 ± 0.1 m/s, which was similar to the speed on the treadmill. Single-sample t-tests revealed that there was a reduction in foot clearance of 0.03 ± 0.01 m (t = − 10.28, *p* < 0.001) from BASE to TF on Day 1 and this corresponded to a relative transfer of 32% (interquartile ratio (IQR) 16%, Wilcoxon signed rank test, z = 3.82, p < 0.001). Additionally, we tested for potential within-block improvement during walking over-ground. There was no significant difference in foot clearance when comparing the first trial and last trial during BASE (t(180) = 0.21, *p* = 0.83) or during TF (t(180) = 0.78, *p* = 0.43), which indicates that there was no within-block improvement during over-ground trials (Fig. 3c).

### Retention of skilled obstacle negotiation

When participants returned to the lab 24 h later, they generally retained the level of performance they achieved at the end of Day 1 (Fig. 2). The average number of collisions was 2 ± 2 out of 40 obstacles. Single-sample t-tests demonstrated that there was a small, but significant increase in foot clearance of 0.008 ± 0.01 m (t = 2.57, *p* = 0.02) from END_VR on Day 1 to RET_VR on Day 2, which indicates that there was some forgetting of the skill. However, there was no significant change in clearance from TF on Day 1 to RET_OG on Day 2, which indicates that this skill was retained after a 24-h retention interval (Fig. 3b). There was no significant difference between the first and the last trials during RET_OG (t(180) = 1.13, *p* = 0.26), which indicates that participants sustained the level of performance throughout the over-ground trials on Day 2 (Fig. 3c).

### Prediction of retention in VR and over-ground

**Fig. 4:**
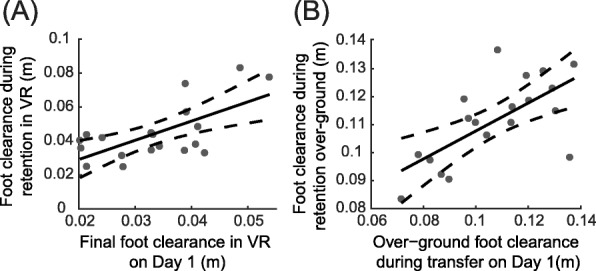
Associations between performance on Day 1 and retention on Day 2. **a** Relationship between foot clearance during retention in VR on Day 2 (RET_VR) and the final foot clearance in VR on Day 1. **b** Relationship between over-ground foot clearance during retention on Day 2 (RET_OG) and over-ground foot clearance during transfer (TF) on Day 1. Each participant is represented by a single data point, the solid black line represents the regression fit, and the dashed gray lines are 95% confidence intervals

After obtaining individual model parameters *A*_*i*_, *tau*_*i*_, and *D*_*i*_, we then calculated acquisition-related variables (Table 1) to identify potential predictors of performance during retention. The final model with the lowest BIC included only the estimated final foot clearance during acquisition as a significant predictor of retention in VR (Adjusted R^2^ = 0.40, t = 3.60, *p* = 0.002, β = 1.14, standard error (SE) = 0.33, Fig. 4a). Participants who achieved lower foot clearance at the end of Day 1 were more likely to maintain a low foot clearance on Day 2. This association indicates that the performance at the end of practice is an important predictor of 24-h retention.

Variable selection for the model of over-ground retention demonstrated that foot clearance during transfer to over-ground on Day 1 was the only significant predictor of retention in over-ground (Adjusted R^2^ = 0.42, t = 3.72, p = 0.002, β = 0.50, SE = 0.13, Fig. 4b). Specifically, lower clearance during RET_OG was associated with lower foot clearance during TF on Day 1. Together, the results suggest that the degree of transfer to a new context on Day 1 is an important predictor of how performance in that context is retained 24 h later.

## Discussion

The objectives of this study were to determine how individual differences in obstacle negotiation skill acquisition in VR influence retention and transfer to over-ground walking. We found that our participants successfully reduced foot clearance as instructed during practice trials, transferred the reduced foot clearance to over-ground obstacle negotiation, and retained the reduced foot clearance after 24 h. Furthermore, retention in each environment was associated with measures of performance during practice in the same environment. Together, our results demonstrate that locomotor skills can be learned in VR and that measures of performance during skill acquisition predict retention in a context-dependent manner.

A previous study that used the same instruction to minimize clearance during physical obstacle negotiation found that participants reduced their foot clearance by about 40% during a single day of acquisition [31]. Based on our NLME results, foot clearance during virtual obstacle negotiation in VR was reduced by a mean of 69 ± 15% during locomotor skill acquisition on Day 1. Further, we found evidence of forgetting after a 24-h retention period, but the performance decrement was small.

The similarity in performance improvement in physical training from the previous study [31] and virtual training from our study suggests that obstacle negotiation skills are learned similarly in VR and the real world. However, the absolute initial and final foot clearance during practice were higher in our study than the previous study. Initial and final foot clearance in the van Hedel and Dietz study was approximately 5 cm and 2 cm, respectively while in our study was approximately 13 cm and 4 cm, respectively. The exaggerated foot clearance in VR may be due to the overestimation of obstacle height as individuals tend to overestimate height in VR [38]. This over-estimation may limit the degree to which participants are capable of reducing foot clearance to avoid a collision. Nevertheless, these results suggest that VR has the potential to be used as a locomotor skill learning tool that can facilitate skill transfer and retention in the real world.

### Transfer and retention of locomotor skills learned in virtual reality

In line with our hypothesis, the reduction in foot clearance in VR during practice on Day 1 was transferred to over-ground walking. After practicing in VR, individuals reduced their foot clearance during over-ground walking by a mean of 21 ± 9% relative to baseline. When this change was expressed relative to the change in performance from the beginning to the end of practice in VR in a similar manner as Torres-Oviedo and Bastian [24], this corresponded to a median relative transfer of 32% (IQR 16%). This level of relative transfer was comparable to what has been observed during adaptation to a gradual perturbation during split-belt walking where aftereffects in over-ground walking were ~ 35% of the difference in performance between baseline and catch trials [24]. Together, these results demonstrate that locomotor skills acquired through both implicit adaptive learning, which primarily involves the cerebellum [39, 40], and more explicit skill-based learning, which is more associated with the prefrontal cortex [40, 41], transfer to environments that differ from that in which training occurred. Given that the neural processes underlying these two types of learning differ markedly from one another, the similarity in transfer between these studies may reflect a fundamental feature of how skills learned on a treadmill transfer to over-ground walking.

It would be of interest for future studies to determine how transfer differs following over-ground VR-based training relative to when VR training is implemented on a treadmill. This could help further establish which features of the training environment are most important for promoting transfer of learning to the real world.

Our results are not consistent with those from a recent study of skilled upper extremity motor learning which did not transfer from VR to the real world [42]. When participants acquired a sequential isometric pinch task in VR, subsequent performance of the same task in the real world was significantly slower and approximately 29% less accurate than performance in the last block of practice in VR, suggesting that there was a decrement of performance from VR to the real world. However, this discrepancy may stem from differences in our measure of transfer. Here we calculated transfer as the difference in performance from a baseline over-ground trial, which occurred before practice and a post-practice trial which also occurred over-ground. In contrast, Anglin and colleagues calculated transfer as the difference in performance between the end of practice in VR and a subsequent practice trial in the real world. Therefore, their results may reflect aspects of transfer of skill acquisition, context-dependent differences in performance, or both. Future studies should record baseline performance in each context to assess transfer of motor skills from VR to the real world.

The reduction in foot clearance on Day 2 was generally retained in VR and over-ground relative to the last block of each environment on Day 1. Although we observed significant forgetting of the locomotor skill in VR after 24 h, the increase in foot clearance on Day 2 compared to the end of practice was only 8 mm. Given that the reduction in foot clearance during skill acquisition was 9 cm, an 8 mm performance decrement can be considered negligible. Studies of split-belt treadmill adaptation consistently reported a ~ 80% reduction in step length asymmetry after multiple days of practice [14–16]. Moreover, individuals who learned to track a target during the swing phase of gait retained approximately an 38% reduction in tracking error compared to baseline on Day 1 [20]. We observed a 59% reduction of foot clearance from baseline to retention in VR. These results demonstrate that locomotor skills can be acquired and retained following multiple forms of practice including treadmill-based VR training.

We also found that there were no changes in foot clearance during over-ground retention relative to the over-ground transfer trial on Day 1. There are two potential explanations for this observation. First, this may reflect the fact that retention during over-ground walking was comparable to retention in VR. Second, it is possible that some of the observed retention during over-ground walking on Day 2 may have resulted from the additional practice performed during the retention block in VR as this was always tested before retention in over-ground. These potential explanations could be disambiguated in future studies where the evaluation of retention in the non-trained environment and retention in VR are counterbalanced.

When considering VR-based rehabilitation, however, it is important to note that aging and pathology can affect the level of retention and sustained transfer. Learning impairments could result from neurodegenerative changes such as atrophy of prefrontal gray matter or impairment of the frontostriatal network, which are important in motor learning [43–46]. For example, Parkinson’s disease may reduce an individual’s ability to transfer learned motor skills due to declines in executive functions such as working memory and cognitive flexibility or an inability to form accurate motor memories of the task [47].

### Performance in each environment was a strong predictor of retention in the same environment

The level of foot clearance measured during retention in VR on Day 2 was strongly associated with the final foot clearance during acquisition in VR such that participants who achieved lower clearance at the end of acquisition also achieved lower clearance during retention. In contrast to previous literature where the amount and rate of performance change during skill acquisition predicted motor skill learning [25–27], neither the amount nor the rate of skill acquisition were important predictors of retention in VR on Day 2. Although previous studies did not explicitly investigate performance at the end of the practice block as a potential predictor, final performance may have been associated with other significant predictors found in previous studies such as the amount and rate of skill acquisition. Other work has shown that the level of performance during acquisition is not always related to performance during retention [21, 48–50]. For instance, Schmidt and Djork (1992) found that features of practice that impaired performance during acquisition such as a random practice schedule, reduced feedback frequency, or contextual interference enhanced long-term retention [48]. Understanding how the practice structure used during VR-based training influences long-term retention and transfer is an important area of future study.

Retention of foot clearance during over-ground walking on Day 2 was also associated with foot clearance during the over-ground transfer block, which is equivalent to final foot clearance over-ground on Day 1. Individuals who achieved lower foot clearance during transfer on Day 1 exhibited lower foot clearance during retention on Day 2. This is consistent with the primary predictor of retention in VR and together provides evidence that the final level of performance during practice is an important predictor of retention. Given the moderate amount of variance accounted for by our models of retention performance, there are likely important, potentially subject-specific, factors such as variance in the amount of forgetting between days that also influence retention performance. Overall, the context-specificity of predictors of retention suggests that different memory processes may underlie the expression of the learned locomotor skills in differing contexts. Therefore, measures of transfer in addition to retention are important to fully predict lasting effects of VR training on locomotor performance.

## Conclusion

Retention and transfer of motor skills are integral features of motor skill learning and form the basis by which training in virtual environments can impact real-world behavior. Here, we demonstrated that individuals could acquire a strategy for skilled obstacle negotiation in VR, transfer the skill to over-ground walking, and retain the skill after 24 h. Moreover, the extent of retention and transfer was predicted by individual differences in the final performance in a context-dependent manner. Specifically, performance measures in each environment were strongly correlated with retention performance in the same environment. Overall, our findings support the use of VR as an effective tool to train skilled obstacle negotiation and facilitate the transfer of improvements in obstacle negotiation to the real-world.

## Data Availability

The data collected during this study will be provided upon request made to the corresponding author.

## Abbreviations

BASE: Baseline during over-ground walking
BASE_VR: Baseline in virtual reality
END_VR: End of practice block (block 3) in virtual reality
*FC*: Estimated foot clearance in virtual reality
HMD: Head-mounted display
NLME: Nonlinear mixed-effects
RET_OG: Retention during over-ground walking
RET_VR: Retention in virtual reality
TF: Transfer during over-ground walking
VIF: Variance Inflation Factor
VR: Virtual reality

## References

[1] Jaffe DL, Brown DA, Pierson-Carey CD, Buckley EL, Lew HL (2004). Stepping over obstacles to improve walking in individuals with poststroke hemiplegia. J Rehabil Res Dev.

[2] Rizzo A, Kim GJ (2005). A SWOT analysis of the field of virtual reality rehabilitation and therapy. Presence Teleoper Virtual Env.

[3] Fung J, Richards CL, Malouin F, McFadyen BJ, Lamontagne A (2006). A treadmill and motion coupled virtual reality system for gait training post-stroke. Cyberpsychology Behav Impact Internet Multimed Virtual Real Behav Soc.

[4] Yang Y-R, Tsai M-P, Chuang T-Y, Sung W-H, Wang R-Y (2008). Virtual reality-based training improves community ambulation in individuals with stroke: a randomized controlled trial. Gait Posture.

[5] Mirelman A, Maidan I, Herman T, Deutsch JE, Giladi N, Hausdorff JM (2011). Virtual reality for gait training: can it induce motor learning to enhance complex walking and reduce fall risk in patients with Parkinson’s disease?. J Gerontol A Biol Sci Med Sci.

[6] Mirelman A, Rochester L, Maidan I, Del Din S, Alcock L, Nieuwhof F, et al. Addition of a non-immersive virtual reality component to treadmill training to reduce fall risk in older adults (V-TIME): a randomised controlled trial. Lancet. 2016. 10.1016/S0140-6736(16)31325-3 27524393

[7] Shema SR, Brozgol M, Dorfman M, Maidan I, Sharaby-Yeshayahu L, Malik-Kozuch H (2014). Clinical experience using a 5-week treadmill training program with virtual reality to enhance gait in an ambulatory physical therapy service. Phys Ther.

[8] Parijat P, Lockhart TE, Liu J (2015). Effects of perturbation-based slip training using a virtual reality environment on slip-induced falls. Ann Biomed Eng.

[9] Stolze H, Klebe S, Zechlin C, Baecker C, Friege L, Deuschl G (2004). Falls in frequent neurological diseases--prevalence, risk factors and aetiology. J Neurol.

[10] Maagaard M, Sorensen JL, Oestergaard J, Dalsgaard T, Grantcharov TP, Ottesen BS (2011). Retention of laparoscopic procedural skills acquired on a virtual-reality surgical trainer. Surg Endosc.

[11] Ghanbarzadeh Reza, Ghapanchi Amir Hossein, Blumenstein Michael, Talaei-Khoei Amir (2014). A Decade of Research on the Use of Three-Dimensional Virtual Worlds in Health Care: A Systematic Literature Review. Journal of Medical Internet Research.

[12] Siu K-C, Best BJ, Kim JW, Oleynikov D, Ritter FE (2016). Adaptive virtual reality training to optimize military medical skills acquisition and retention. Mil Med.

[13] Vaughan N, Gabrys B, Dubey VN (2016). An overview of self-adaptive technologies within virtual reality training. Comput Sci Rev.

[14] Malone LA, Vasudevan EVL, Bastian AJ (2011). Motor adaptation training for faster relearning. J Neurosci.

[15] Leech KA, Day KA, Roemmich RT, Bastian AJ (2018). Movement and perception recalibrate differently across multiple days of locomotor learning. J Neurophysiol.

[16] Day KA, Leech KA, Roemmich RT, Bastian AJ (2018). Accelerating locomotor savings in learning: compressing four training days to one. J Neurophysiol.

[17] Fortin K, Blanchette A, McFadyen BJ, Bouyer LJ (2009). Effects of walking in a force field for varying durations on aftereffects and on next day performance. Exp Brain Res.

[18] Gordon KE, Ferris DP (2007). Learning to walk with a robotic ankle exoskeleton. J Biomech.

[19] Smith JD, Martin PE (2007). Walking patterns change rapidly following asymmetrical lower extremity loading. Hum Mov Sci.

[20] Krishnan C, Washabaugh EP, Reid CE, Althoen MM, Ranganathan R (2018). Learning new gait patterns: age-related differences in skill acquisition and interlimb transfer. Exp Gerontol.

[21] Schmidt R, Lee T (2011). Motor control and learning: a behavioral emphasis.

[22] Cothros N, Wong JD, Gribble PL (2006). Are there distinct neural representations of object and limb dynamics?. Exp Brain Res.

[23] Kluzik J, Diedrichsen J, Shadmehr R, Bastian AJ (2008). Reach adaptation: what determines whether we learn an internal model of the tool or adapt the model of our arm?. J Neurophysiol.

[24] Torres-Oviedo G, Bastian AJ (2012). Natural error patterns enable transfer of motor learning to novel contexts. J Neurophysiol.

[25] Wadden KP, Asis KD, Mang CS, Neva JL, Peters S, Lakhani B (2017). Predicting motor sequence learning in individuals with chronic stroke. Neurorehabil Neural Repair.

[26] Schaefer SY, Duff K. Rapid responsiveness to practice predicts longer-term retention of upper extremity motor skill in non-demented older adults. Front Aging Neurosci. 2015;7. 10.3389/fnagi.2015.00214 PMC4649025 26635601

[27] Park H, Schweighofer N (2017). Nonlinear mixed-effects model reveals a distinction between learning and performance in intensive reach training post-stroke. J Neuroengineering Rehabil.

[28] Chase C, Seidler R (2008). Degree of handedness affects intermanual transfer of skill learning. Exp Brain Res.

[29] Lefumat HZ, Vercher J-L, Miall RC, Cole J, Buloup F, Bringoux L (2015). To transfer or not to transfer? Kinematics and laterality quotient predict interlimb transfer of motor learning. J Neurophysiol.

[30] Erni T, Dietz V (2001). Obstacle avoidance during human walking: learning rate and cross-modal transfer. J Physiol.

[31] van Hedel HJA, Dietz V (2004). The influence of age on learning a locomotor task. Clin Neurophysiol Off J Int Fed Clin Neurophysiol.

[32] Kim A, Kretch KS, Zhou Z, Finley JM. The quality of visual information about the lower extremities influences visuomotor coordination during virtual obstacle negotiation. J Neurophysiol. 2018. 10.1152/jn.00931.2017 PMC6139439 29742030

[33] Krell J, Patla AE (2002). The influence of multiple obstacles in the travel path on avoidance strategy. Gait Posture..

[34] Michel J, Benninger D, Dietz V, van Hedel HJA (2009). Obstacle stepping in patients with Parkinson’s disease. Complexity does influence performance. J Neurol.

[35] Newell KM, Liu YT, Mayer-Kress G (2001). Time scales in motor learning and development. Psychol Rev.

[36] Winter B, Wieling M (2016). How to analyze linguistic change using mixed models, growth curve analysis and generalized additive modeling. J Lang Evol.

[37] James G, Witten D, Hastie T, Tibshirani R (2013). An introduction to statistical learning: with applications in R.

[38] Asjad NS, Adams H, Paris R, Bodenheimer B. Perception of Height in Virtual Reality: A Study of Climbing Stairs. Proc 15th ACM Symp Appl Percept. New York, NY, USA: ACM; 2018. p. 4:1–4:8.

[39] Morton SM, Bastian AJ (2006). Cerebellar contributions to locomotor adaptations during splitbelt treadmill walking. J Neurosci.

[40] Taylor JA, Ivry RB (2014). Cerebellar and prefrontal cortex contributions to adaptation, strategies, and reinforcement learning. Prog Brain Res.

[41] Destrebecqz A, Peigneux P, Laureys S, Degueldre C, Del Fiore G, Aerts J (2005). The neural correlates of implicit and explicit sequence learning: interacting networks revealed by the process dissociation procedure. Learn Mem.

[42] Anglin J, Saldana D, Schmiesing A, Liew S. Transfer of a skilled motor learning task between virtual and conventional environments. 2017 IEEE Virtual Real VR. 2017. p. 401–2.

[43] Cabeza R (2001). Cognitive neuroscience of aging: contributions of functional neuroimaging. Scand J Psychol.

[44] Voelcker-Rehage C (2008). Motor-skill learning in older adults—a review of studies on age-related differences. Eur Rev Aging Phys Act.

[45] Olson M, Lockhart TE, Lieberman A. Motor learning deficits in Parkinson’s disease (PD) and their effect on training response in gait and balance: a narrative review. Front Neurol. 2019;10. 10.3389/fneur.2019.00062 PMC6374315 30792688

[46] Nieuwboer A, Rochester L, Müncks L, Swinnen SP (2009). Motor learning in Parkinson’s disease: limitations and potential for rehabilitation. Parkinsonism Relat Disord.

[47] Marinelli L, Quartarone A, Hallett M, Frazzitta G, Ghilardi MF (2017). The many facets of motor learning and their relevance for Parkinson’s disease. Clin Neurophysiol Off J Int Fed Clin Neurophysiol..

[48] Schmidt RA, Bjork RA (1992). New conceptualizations of practice: common principles in three paradigms suggest new concepts for training. Psychol Sci.

[49] Guadagnoli MA, Lee TD (2004). Challenge point: a framework for conceptualizing the effects of various practice conditions in motor learning. J Mot Behav.

[50] Wulf G, Lewthwaite R (2016). Optimizing performance through intrinsic motivation and attention for learning: the OPTIMAL theory of motor learning. Psychon Bull Rev.

